# Characterization of Pediatric Seizures in the Commonwealth of the Northern Mariana Islands

**DOI:** 10.3390/children7040026

**Published:** 2020-04-01

**Authors:** Ahana Yogesh, Michael Taylor, Mary P. Chang

**Affiliations:** 1 Department of Emergency Medicine, University of Texas Southwestern Medical Center, 5323 Harry Hines Blvd, Dallas, TX 75390, USA; Ahana.Yogesh@UTSouthwestern.edu; 2 Department of Pediatrics, Commonwealth Healthcare Corporation, Garapan, Saipan 96950, Northern Mariana Islands; Michael.K.Taylor@gmail.com

**Keywords:** Pediatric, children, seizure, epilepsy, epidemiology, Saipan

## Abstract

Saipan is a United States (US) territory Western Pacific island where little recent data exists regarding epidemiology, clinical presentation, and standard of care for pediatric seizures. This paper characterizes these features in Saipan’s pediatric population with comparisons to mainland US. This is a retrospective chart review of all pediatric patients with a history of seizures at the island’s only hospital and major private neurology clinic over a 10-year period. Variables regarding demographics, presentation, diagnosis, and treatment were collected. A total of 144 patients were included, with 101 patients diagnosed with febrile seizures and 31 patients diagnosed with non-febrile seizures. Age at first presentation peaked at 1 year old overall. The most common identified etiology of epilepsy was found to be hypoxic injury (39%), hemorrhagic injury (10%), cerebral malformation (6%), and brain mass (6%). Simple versus complex classification of febrile seizures, etiologies, and first-line treatment for non-febrile seizures were comparable to the mainland US. Electroencephalogram (EEG) was not used consistently in diagnosis. The findings from this study demonstrate that clinical presentations of pediatric seizures in Saipan are comparable to those in the mainland US.

## 1. Introduction

While significant data exists describing the epidemiology of childhood seizures in the United States (US), the same cannot be said for other regions in the world. The Commonwealth of the Northern Mariana Islands (CNMI) is a US territory comprised of several islands in the Western Pacific region. Saipan, the capital and the largest of the CNMI islands, has a population of roughly 50,000 people, most of which are Asians and Pacific Islanders [1]. The existing data about pediatric epilepsy that can most closely be approximated to the population of Saipan comes from Guam, a neighboring Mariana Island, and dates back to the early 1970s [2]. Apart from a 2004 World Health Organization publication stating that there was insufficient epidemiological data and unreliable estimates of the epilepsy burden in the Western Pacific region, little more has been published since then [3]. Furthermore, while the Western Pacific islands are close to one another in proximity, the cultural, political, and social nuances of the islands differ greatly, leading to variations in healthcare that warrant exploration. For example, Guam’s population is over three times that of the CNMI and includes a higher ratio of native islanders to Asian immigrants, whereas the CNMI has a roughly equal ratio of the two populations [1]. In this study, we aim to characterize the Saipanese pediatric population and compare to the mainland US. 

## 2. Materials and Methods

This was a retrospective chart review of all patients under the age of 18 years with the ICD−9 and ICD−10 codes corresponding to seizures and epilepsy in the electronic health record system (EHR) at Commonwealth Healthcare Corporation (CHC) and paper charts at a private neurology clinic in Saipan between 2008 to 2018. ICD−9 codes 780.3 (convulsions), 345 (epilepsy and recurrent seizures), 779.0 (convulsions in newborns), and 649.41 (epilepsy complicating pregnancy, childbirth, or the puerperium, delivered, with or without mention of antepartum condition), as well as ICD−10 codes G40 (epilepsy and recurrent seizures), R56 (convulsions, not elsewhere classified), and P90 (convulsions of newborn) were utilized to generate a list of patients who had been diagnosed with seizures or epilepsy. There were 39 patients that did not have adequate data in their electronic or paper records and were subsequently excluded from the study. Patients who were seen both at CHC and the private neurology clinic were only counted once for the purpose of the study. CHC is the only hospital on the island, and the neurology clinic is open for two weeks every year when a neurologist visits Saipan to take care of complex neurology patients. 

Febrile seizures were defined as seizures that occur in febrile children between the ages of 6 and 60 months who do not have an intracranial infection, metabolic disturbance, or history of afebrile seizures [4]. Epilepsy was defined as one of the following: a) at least two unprovoked (or reflex) seizures occurring more than 24 hours apart; b) one unprovoked (or reflex) seizure and a probability of further seizures similar to the general recurrence risk after two unprovoked seizures occurring over the next 10 years; or c) diagnosis of an epilepsy syndrome [5]. Epilepsy was not sub-categorized into absence versus non-absence in this study

The following variables were collected from patient charts: Age, sex, ethnicity, gestational age at birth, history of febrile seizure, age at first presentation, method of diagnosis, etiology of seizure disorder, initial treatment regimen, and presence of off-island referral for seizure disorder. The study was conducted in accordance with the Declaration of Helsinki, and the protocol was approved by the University of Texas at Southwestern Medical Center Institutional Review Board (STU−2018−0222).

## 3. Results

A total of 101 patients with an ICD−9 or ICD−10 code corresponding to febrile convulsions, and 31 patients with an ICD−9 or ICD−10 code corresponding to non-febrile convulsions were included in the study. Of the former group, none were patients of the private neurology clinic; of the latter group, seven were patients of the private neurology clinic. 

### 3.1. Febrile Seizures

**Figure 1:**
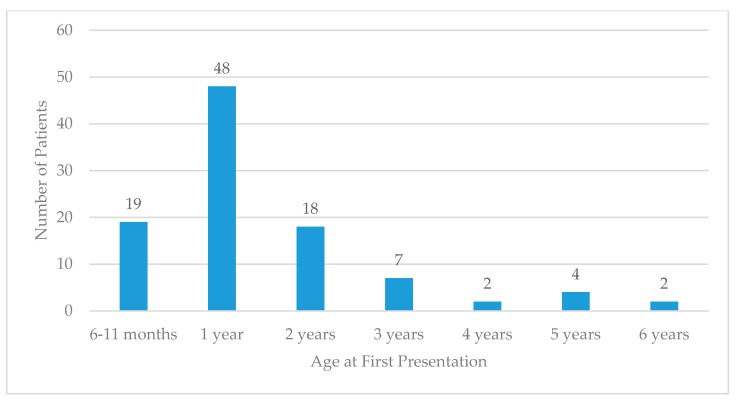
Age at first presentation in Commonwealth Healthcare Corporation (CHC) pediatric patients with febrile seizures.

A total of 61% of patients who met criteria for febrile seizures were male, and 39% were female. Of these patients, 41% identified as native ethnicity (Chamorro or Carolinian) or other Pacific Islander, while 30% were of Asian ethnicity. The majority of patients in this group were diagnosed with simple febrile seizures (Table 1). Age at first presentation peaked at 1 year and declined thereafter (Figure 1).

### 3.2. Non-Febrile Seizures

**Figure 2:**
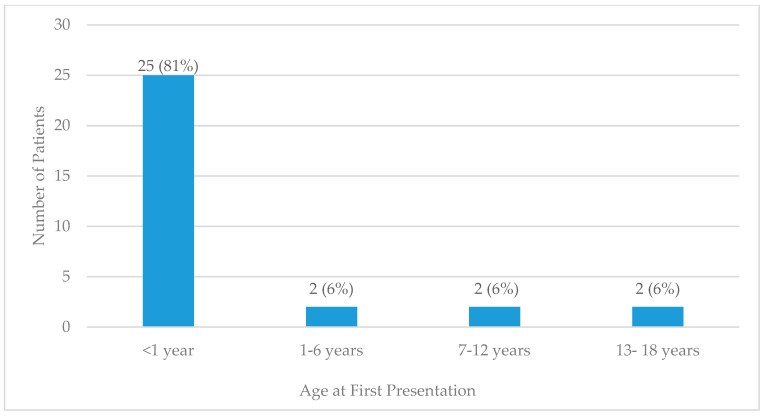
Age at first presentation in CHC pediatric patients with epilepsy.

Of the patients who met criteria for epilepsy, 58% were male and 42% were female. Moreover, 45% were of native ethnicity or other Pacific Islander, and 26% were of Asian ethnicity (Table 1). The majority of patients who met criteria for epilepsy had their first presentation under 1 year of age (81%), most frequently in the neonatal (under 1 month of age) period (Figure 2). 

**Figure 3:**
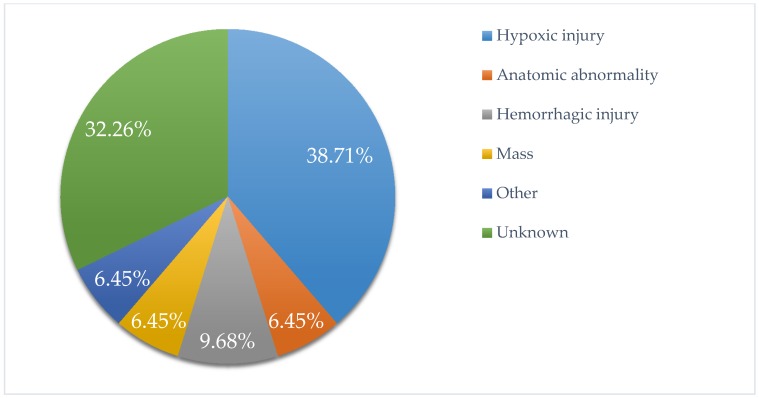
Etiology of seizures in CHC pediatric patients with epilepsy.

The most commonly identified etiology of epilepsy was found to be hypoxic injury (39%), followed by hemorrhagic injury (10%), cerebral malformation (6%), and brain mass (6%). The etiology was not identified or documented in 32% of these patients (Figure 3). 

Electroencephalogram (EEG) was the most common modality of imaging used during work-up, with 72% of patients receiving an EEG at some point in time. Other utilized modalities included computed tomography (CT), head ultrasound (HUS), magnetic resonance imaging (MRI), and lumbar puncture (LP) in descending order of usage (Table 2).

Patients diagnosed with epilepsy most commonly received phenobarbital as initial treatment. Of those who presented with non-febrile seizures, 33% were referred off-island for management.

## 4. Discussion

Overall, in terms of epidemiology and standards of care, we found that the population of children with febrile seizures and epilepsy in Saipan is highly comparable to that of the US. In looking specifically at febrile seizures, we found that the male to female ratio, peak age of presentation, and predominance of simple febrile seizures versus complex febrile seizures are similar to characteristics of febrile seizures in the mainland [6]. The known infectious causes of febrile seizures and epilepsy include viral, bacterial, parasitic, fungal, and prion infections of the central nervous system due to suspected cytokine-mediated inflammatory response, cortical involvement, and brain maturity [7]. There was little documentation of the exact cause of febrile seizures or epilepsy in our patient population, which could be investigated further. Future research exploring the rate of epilepsy development in those with a history of febrile seizures here could help further define similarities and differences between this population in Saipan versus that of the mainland. 

With regards to non-febrile seizures and childhood epilepsy, we found the peak age of presentation to be similar to that of the US, with the majority of patients initially presenting before the age of 1 [8]. For the diagnosis of epilepsy, there was a higher percentage of male children in this population, which is similar to other epidemiological studies worldwide [9]. Breakdown of etiologies of epilepsy also paralleled major etiologies nationally [10]. More specifically, the percentage of non-febrile seizures thought to be of infectious etiology was less than 1%, compared to 3% nationally. This was an unexpected finding given the prevalence of certain infectious etiologies in tropical and/or developing regions, including malaria and neurocysticercosis [11]. This suggests that providers at CHC can expect similar profiles of childhood epilepsy cases as they would if practicing on the mainland. 

Interestingly, we found that fewer than 75% of patients that presented with non-febrile seizures received an EEG. The majority of patients (92%) that did not receive an EEG had their initial presentation in the neonatal period. This, in contrast to previously discussed findings, represents a deviation from standards of care in the US, where EEG screening is routinely done for all patients presenting with seizures in the neonatal period, per American Academy of Neurology (AAN) recommendations [12]. Currently, CHC does not have an EEG machine as part of the hospital facilities, and all patients with seizures must be referred to a specific private clinic on the island with a working machine. Once patients are referred, make an appointment, and undergo EEG, the results must be sent off-island for interpretation, after which CHC providers must schedule follow-up appointments with patients to discuss the results. Based on chart review, there were also several occasions during which the EEG machine was not usable, and the patient was subsequently lost to follow-up. Unfortunately, this system increases the likelihood that patients may be lost to follow-up or face delays in appropriate treatment. Additionally, research has found that some high-risk neonatal populations, such as those at risk for or those who have already demonstrated acute brain injury, often present exclusively with subclinical seizures [13]. Without EEG monitoring in these patients, subclinical seizures can go unrecognized, leaving a portion of the patient population at risk for untreated seizure activity. Given the percentage of CHC’s epileptic patients who present in the neonatal period, having an EEG machine for the hospital will help mitigate delays to obtaining efficient and time-sensitive interpretation by off-island epileptologists. Of note, it is possible that the 28% of our childhood epilepsy population who did not receive EEG is an over-estimation, given factors such as poor documentation in charts and missing information from off-island referrals. Consistent documentation of both on- and off-island data in the EHR will likely allow for more accurate estimation of EEG utilization in the future. With respect to imaging, CT and HUS were the next most commonly used modalities, given that there is no MRI machine available on the island. This is in accordance with guidelines for seizure work-up when MRI is not available, and CT and US should continue to be used for patients who are unable to secure off-island referral [14]. In some seizure types, such as absence seizures, using multiple imaging modalities is helpful to detect where the subcortical focus of the seizures is located. Simultaneous EEG recording with either functional magnetic resonance imaging, positron-emission tomography, low resolution electromagnetic tomography, single photon emission spectroscopy, near-infrared spectroscopy, or optical imaging of intrinsic signals can help provide some spatial mapping of the epileptic foci [15]. Patients in Saipan would have to travel off the island to have these studies completed. 

Also in accordance with national standards of care is the first-line treatment for non-febrile seizures with phenobarbital [16]. The World Health Organization 2011 review found the benefits of treatment with short term anti-epileptic drugs (AEDs) outweighed potential risks [17]. Interestingly, emerging research looking into ideal treatment duration with AEDs has suggested that early discontinuation of phenobarbital, potentially at time of hospital discharge, may not be harmful [17]. The sedating effects of phenobarbital, can interfere with establishment of oral feeding regimens and long-term brain development, suggesting that the risks of continuing medication post-discharge may outweigh the benefits [18,19,20]. If these findings are validated, CHC would benefit greatly from following the practice of terminating AED use at time of hospital discharge due to observations of inconsistent AED use in this population. While a several month-long phenobarbital taper was commonly prescribed, it was noted during chart review that the actual duration of time a patient was on an AED was subject to variation and not always consistent with the prescribed duration. For example, patients would often return for their three-month follow-up having stopped their AED weeks ago, whereas other patients would delay their follow-up appointments and request refills in the meantime. This seemed to be a common issue in this patient population, which raises the issue of over- and under-treatment. In the future, guidelines promoting AED cessation at time of discharge may serve this population well. In the meantime, based on preliminary research, the ideal duration of AED use should be determined by both duration of seizures and by need for a second AED to control seizure activity [21].

Another goal of this research was to comment on the potential need for a full-time pediatric neurologist on island. Currently, a neurologist from the mainland makes yearly trips to Saipan for two weeks a time to see patients who have been referred from CHC or other private clinics. This neurologist also offers remote consultation for both existing and newly referred patients during the remainder of the year. While having a pediatric neurologist on island full-time would offer continuity of care and the ability to provide more efficient and time-sensitive treatment, the patient burden is far too low for this to be warranted. Of the 31 patients in our study who were, at one point, diagnosed with epilepsy, only nine were referred to and seen by the neurologist during a recent visit, suggesting that several of the patients with childhood epilepsy may not even require treatment at this point, or may have found providers elsewhere. We also found that several of the patients referred off-island for seizure management also had several other multi-system medical issues that demanded care from specialists who do not practice in Saipan, suggesting that several of these patients would require off-island referrals regardless of the presence of a pediatric neurologist.

While we took several measures to prevent and adjust for biases during this study, it is important to note some key limitations. The most prominent barrier to accurate data collection was incomplete data documentation. This also includes patients who were referred off-island but did not have records transmitted back to CHC, and those who may have been diagnosed in Saipan but moved away. Therefore, results in several categories are likely under-represented. This issue was further complicated by the recent conversion from paper to electronic charts at CHC, a transition that was accompanied by loss of records and difficulty accessing all the information in a single patient’s chart. These factors likely all contributed to the introduction of a measurement bias. Additionally, we were only able to collect data from CHC and a major private clinic, and while these populations do likely represent the majority of pediatric patients with seizures on island, there is still missing information from all patients at other private clinics. Finally, our data was limited to patients living in Saipan and those referred to CHC from the neighboring islands of Tinian and Rota. Future studies could explore populations in nearby Micronesia to expand on our findings and increase generalizability. 

## 5. Conclusions

To summarize, we found that amongst a representative pediatric population in Saipan, clinical presentations, etiologies and standards of treatment, were largely similar to those in the mainland US, with a few differences. Prior to this research, trends and heuristics in Saipan regarding pediatric patients with seizures were largely based on anecdotal evidence. The findings from this study serve to inform CHC providers, a majority of whom completed their training in the mainland, that the clinical presentation of pediatric seizures in Saipan largely mirrors that of the US. We also identified an important barrier to care and a key difference between standard of care in Saipan and in the mainland, which is EEG utilization. In order to ensure consistent EEG usage in patients with first-time seizures and facilitate individualized diagnosis and treatment, the hospital would need to obtain an EEG machine. This study also provides data for the consideration of the need for a pediatric neurologist on island. Based on patient volume and multi-system comorbidities that may warrant off-island referral in our population of interest, a full time pediatric neurologist may not be needed. In the future, research delineating rates of seizure freedom in this population with childhood epilepsy may offer further insight into the burden of seizures in Saipan. 

## Figures and Tables

**Table 1:** Demographics and seizure history in Commonwealth Healthcare Corporation (CHC) pediatric patients.

Variable	Patients with Febrile Seizures (FS)	Patients with Non-Febrile Seizures (NFS)
Total, *n*	101	31
Male, *n* (%)	62 (61)	18 (58)
Female, *n* (%)	39 (39)	13 (42)
Native ethnicity or other Pacific Islander, *n* (%)		
	41 (41)	14 (45)
Asian ethnicity, *n* (%)	30 (30)	8 (26)
Delivered at term, *n* (%)	62 (61)	17 (55)
Not delivered at term, *n* (%)	13 (13)	11 (36)
Categorized as simple FS, *n* (%)	97 (96)	*n*/a
Categorized as complex FS, *n* (%)	4 (4)	*n*/a

**Table 2:** Methods of diagnoses in patients with non-febrile seizures.

Modality, N (%)	Patients with Non-Febrile Seizures
EEG, *n* (%)	31 (72)
CT, *n* (%)	24 (56)
HUS, *n* (%)	27 (38)
MRI, *n* (%)	13 (30)
LP, *n* (%)	7 (16)

Abbreviations: EEG = electroencephalogram, CT = computed tomography, HUS = head ultrasound, MRI = magnetic resonance imaging, LP = lumbar puncture.
